# The mapping of mRNA alterations elucidates the etiology of radiation-induced pulmonary fibrosis

**DOI:** 10.3389/fgene.2022.999127

**Published:** 2022-10-24

**Authors:** Meng Yuan, Maoyuan Zhao, Xin Sun, Zhouguang Hui

**Affiliations:** ^1^ Department of Radiation Oncology, National Cancer Center/National Clinical Research Center for Cancer/Cancer Hospital, Chinese Academy of Medical Sciences and Peking Union Medical College, Beijing, China; ^2^ Department of VIP Medical Services, National Cancer Center/National Clinical Research Center for Cancer/Cancer Hospital, Chinese Academy of Medical Sciences and Peking Union Medical College, Beijing, China

**Keywords:** mRNA, radiation-induced pulmonary fibrosis, mouse model, thoracic radiotherapy, RNA sequencing

## Abstract

The etiology of radiation-induced pulmonary fibrosis is not clearly understood yet, and effective interventions are still lacking. This study aimed to identify genes responsive to irradiation and compare the genome expression between the normal lung tissues and irradiated ones, using a radiation-induced pulmonary fibrosis mouse model. We also aimed to map the mRNA alterations as a predictive model and a potential mode of intervention for radiation-induced pulmonary fibrosis. Thirty C57BL/6 mice were exposed to a single dose of 16 Gy or 20 Gy thoracic irradiation, to establish a mouse model of radiation-induced pulmonary fibrosis. Lung tissues were harvested at 3 and 6 months after irradiation, for histological identification. Global gene expression in lung tissues was assessed by RNA sequencing. Differentially expressed genes were identified and subjected to functional and pathway enrichment analysis. Immune cell infiltration was evaluated using the CIBERSORT software. Three months after irradiation, 317 mRNAs were upregulated and 254 mRNAs were downregulated significantly in the low-dose irradiation (16 Gy) group. In total, 203 mRNAs were upregulated and 149 were downregulated significantly in the high-dose irradiation (20 Gy) group. Six months after radiation, 651 mRNAs were upregulated and 131 were downregulated significantly in the low-dose irradiation group. A total of 106 mRNAs were upregulated and 4 downregulated significantly in the high-dose irradiation group. Several functions and pathways, including angiogenesis, epithelial cell proliferation, extracellular matrix, complement and coagulation cascades, cellular senescence, myeloid leukocyte activation, regulation of lymphocyte activation, mononuclear cell proliferation, immunoglobulin binding, and the TNF, NOD-like receptor, and HIF-1 signaling pathways were significantly enriched in the irradiation groups, based on the differentially expressed genes. Irradiation-responsive genes were identified. The differentially expressed genes were mainly associated with cellular metabolism, epithelial cell proliferation, cell injury, and immune cell activation and regulation.

## 1 Introduction

Radiotherapy is one of the primary treatments for cancer. More than two-thirds of all patients with cancer receive radiotherapy throughout the treatment procedure. Radiation-induced lung injury, which includes radiation-induced pneumonitis and pulmonary fibrosis, is a common and serious dose-limiting toxicity of thoracic radiotherapy and can be life-threatening. Radiation-induced pulmonary fibrosis (RIPF) is a late radiation-induced lung injury, and is generally incident 3 months post-irradiation, with symptoms ranging from mild dyspnea to chronic pulmonary insufficiency (Graves et al., 2010). Currently, the etiology of RIPF has not yet been established, and effective interventions that can prevent or cure RIPF are still lacking.

According to previous studies, numerous signaling pathways are involved in the initiation and progression of RIPF. TGF-β is a multifunctional regulator of epithelial-mesenchymal transition, and cell growth and differentiation in response to injuries (Border and Noble, 1994; Chapman, 2011). Radiation-induced activation of the TGF-β pathway has been reported to be critically involved in the pathogenesis of RIPF (Rube et al., 2000). Hypoxia-inducible factor-1α, a key transcription factor regulating several genes in response to hypoxic stimuli, can affect irradiation-induced epithelial-mesenchymal transition *via* the TGFβ-R1/Smad3 signaling pathway (Choi et al., 2015). Besides, the activation of CB1 has been reported to exert pro-inflammatory or pro-oxidant effects, further leading to RIPF (Rajesh et al., 2012; Jourdan et al., 2013). Hence, the corresponding mRNAs related to the above mentioned activated pathways and proteins are vital to the etiology of RIPF and potential treatment strategies, and may also act as biomarkers in the diagnosis of RIPF.

In this study, we aimed to identify the genes responsive to irradiation in an RIPF mouse model using high-throughput RNA sequencing, and compare the genome expression between normal lung tissue and lung tissues from irradiated groups to map the mRNA alterations in RIPF. We also performed Gene ontology (GO) and Kyoto Encyclopedia of Genes and Genomes (KEGG) pathway analyses to identify the function of the differentially expressed genes (DEGs) in the RIPF process.

## 2 Methods and materials

### 2.1 Development of the RIPF mouse model

Thirty-six-week-old male C57BL/6 mice were acquired from HFK Bioscience Co., Ltd. (Beijing, China). The mice were raised at the Experimental Animal Center of the Chinese Academy of Medical Sciences. The *in vivo* study protocols were approved by the Institutional Animal Care and Use Committee (NCC 2020A288) of the National Cancer Center/Cancer Hospital, Chinese Academy of Medical Sciences (Beijing, China).

The mice were randomly divided into 3 groups (10 mice per group): control group (no irradiation), low-dose irradiation group, and high-dose irradiation group. The 10 mice in the low-dose group received 16 Gy single-dose thoracic radiation, while the mice in the high-dose group received thoracic radiation of 20 Gy. Radiation was administered using a Varian Unique-SN2242 unit at 300 cGy/min (1 m source to skin distance). The mice were anesthetized using Avertin (300 mg/kg), and were placed, in a supine position, on a fixing device and only the chest was exposed to radiation. Five mice from each group were euthanized 3 months after radiation. The process was repeated 6 months after radiation. After being euthanized, the left lobes of the lung tissues were extracted from the mice, frozen in liquid nitrogen, and stored at −80°C for RNA sequencing, while the right lobes were fixed in 4% formalin for pathological examination. For ethical considerations, mice with a weight loss of ≥20% were euthanized.

### 2.2 Lung histology

To identify histological alterations caused by pulmonary fibrosis, histological analysis was performed as previously described (Abdollahi et al., 2005). The lung tissues of each mouse were fixed in 4% formalin, followed by overnight fixation, and were further embedded in paraffin, sectioned at 5 mm, and stained with hematoxylin and eosin or Masson’s trichrome stain to assess collagen deposition.

### 2.3 cDNA library preparation and RNA sequencing

To compare the gene expression in lung tissues of normal mice and those of irradiated mice, apart from assessing the effects of different radiation doses on gene expression, tissue RNA was extracted and subjected to high-throughput sequencing using Illumina HiSeq. Total RNA was extracted from lung tissue samples using the TRIzol reagent (Invitrogen), according to the manufacturer’s instructions, as previously described (Liu et al., 2011). After cluster generation, the cDNA library preparations were sequenced on the Illumina sequencing platform, and 150 bp paired-end reads were generated.

### 2.4 Quality control and quantification

Clean data were obtained by removing reads containing adapters, N bases, and low-quality reads from the raw data. The Q20, Q30, and GC values were calculated. All downstream analyses were based on high-quality clean data. The index of the reference genome was built using HISAT2 V2.0.5, and it was also used to align clean paired-end reads to the reference genome. We selected HISAT2 as the mapping tool because it can generate a database of splice junctions based on the gene model annotation file, producing better mapping results than other non-splice mapping tools. Feature Counts V1.5.0 was used to count the read numbers mapped to each gene. The expected number of fragments per kilobase of transcript sequence per million base pairs sequenced (FPKM) in each gene was calculated based on the length of the gene and the read count mapped to that gene.

### 2.5 Differential expression and enrichment analyses

Analysis of differentially expressed genes in the low-dose and high-dose irradiation groups was performed using the DESeq2 R package (1.20.0), which provides statistical routines for determining differential expression using a model based on the negative binomial distribution. *p*-values were adjusted using Benjamini and Hochberg’s approach. Genes identified by DESeq2 to have an adjusted *p*-value ˂ 0.05 were denoted as differentially expressed. Enrichment analyses of these differentially expressed genes were performed. We used the clusterProfiler R package for GO function enrichment analysis, KEGG pathway enrichment analysis, and Reactome enrichment analysis of the differential genes.

### 2.6 Evaluation of immune cell infiltration

CIBERSORT is a method for characterizing different types and relative abundances of immune cells in a mixed cell population. To estimate and compare the different proportions of infiltrated immune cells between the low-dose and high-dose irradiation groups, a gene expression matrix was uploaded to the CIBERSORT web portal (http://cibersortx.stanford.edu/) to analyze immune cell infiltration. The unpaired *t*-test was used to compare the infiltration levels of the immune cells in the two groups.

## 3 Results

### 3.1 Lung remodeling after thoracic radiation

**FIGURE 1:**
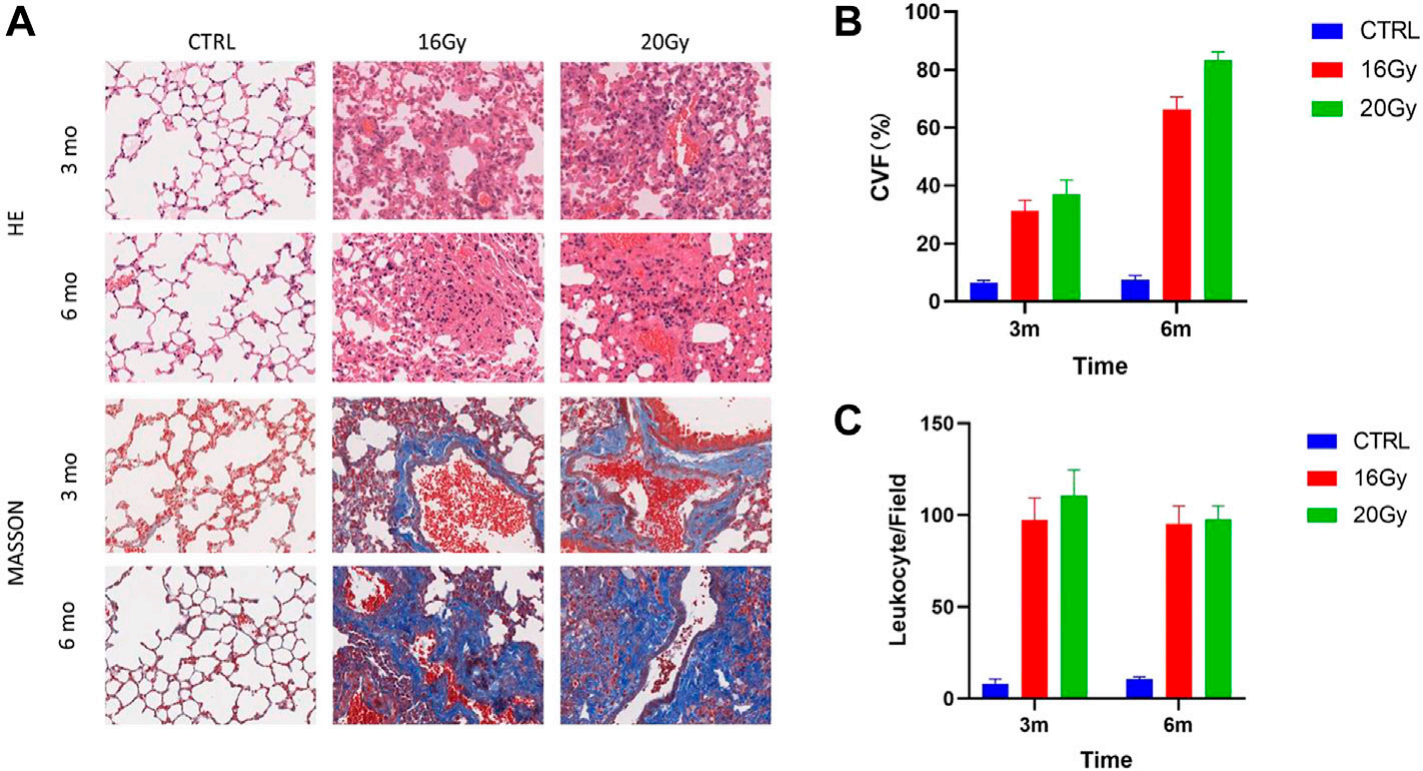
Lung remodeling after thoracic radiation. Thirty mice were randomized into 3 groups: Control group, low-dose irradiation (16 Gy) group and high-dose irradiation (20 Gy) group. Lung tissues were harvested at 3 and 6 months after radiation, for histological identification. **(A)** HE and Masson staining of mouse lung tissues. **(B)** Quantification of septal thickness by analysis of collagen volume fraction. Septal thickness increased at 3 months after radiation and elevated further at 6 months after thoracic irradiation. **(C)** Quantification of leukocyte infiltration. Leukocyte infiltration was significantly increased in the radiation groups. CVF, collagen volume fraction; CTRL, control group.

Pulmonary septal thickness can represent morphological and functional changes during radiation-induced fibrogenesis. The septal thickness increased 3 months after radiation. Extensive lung remodeling occurred within 6 months after thoracic irradiation (Figure 1A). Moreover, the septal thickness in the low-dose and high-dose irradiation groups was severely elevated, compared with that in the control group, corresponding to a strong increase in the interstitial collagen fiber deposition as assessed by Masson staining, consistent with irradiation-induced fibrogenesis (Figure 1B). Additionally, inflammation and immune cell infiltration were significantly increased in the radiation groups (Figure 1C).

### 3.2 RNA sequencing reads quality control

After filtering the raw reads and checking the sequencing error rate and GC content distribution, clean reads for subsequent analyses were obtained. The Q20 was between 95% and 100% and Q30 was between 90% and 95%. The GC content in the clean reads was approximately 50%. The total mapping rate ranged from 96% to 98%, and the unique mapping rate ranged from 91% to 93%.

### 3.3 Gene expression quantification

**FIGURE 2:**
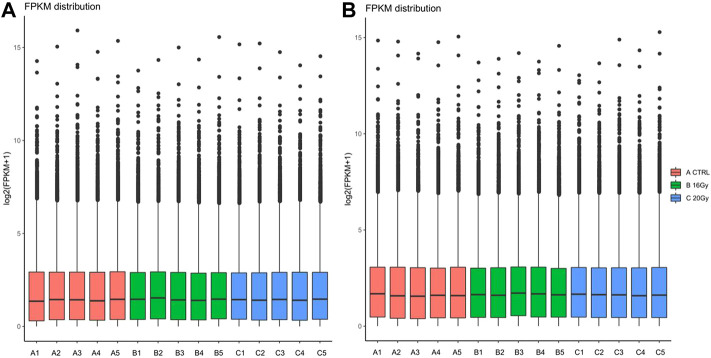
Distribution of gene expression values of mice quantified by FPKM algorithm at **(A)** 3 months after radiation therapy and **(B)** 6 months after radiation therapy. Thirty mice were randomized into 3 groups: control group, low-dose irradiation (16 Gy) group and high-dose irradiation (20 Gy) group. Lung tissues were harvested at 3 and 6 months after radiation to perform RNA sequencing. FPKM, fragments per kilobase of transcript sequence per million base pairs sequenced.

We performed a quantitative analysis of the gene expression levels for each sample and then combined the data to obtain the expression matrix of all the samples. The gene expression level was represented by FPKM, which was adjusted for the sequencing depth and gene length (Bray et al., 2016). The distribution of gene expression levels in the different samples is shown in the boxplot in Figure 2.

### 3.4 Radiation-induced DEGs

**FIGURE 3:**
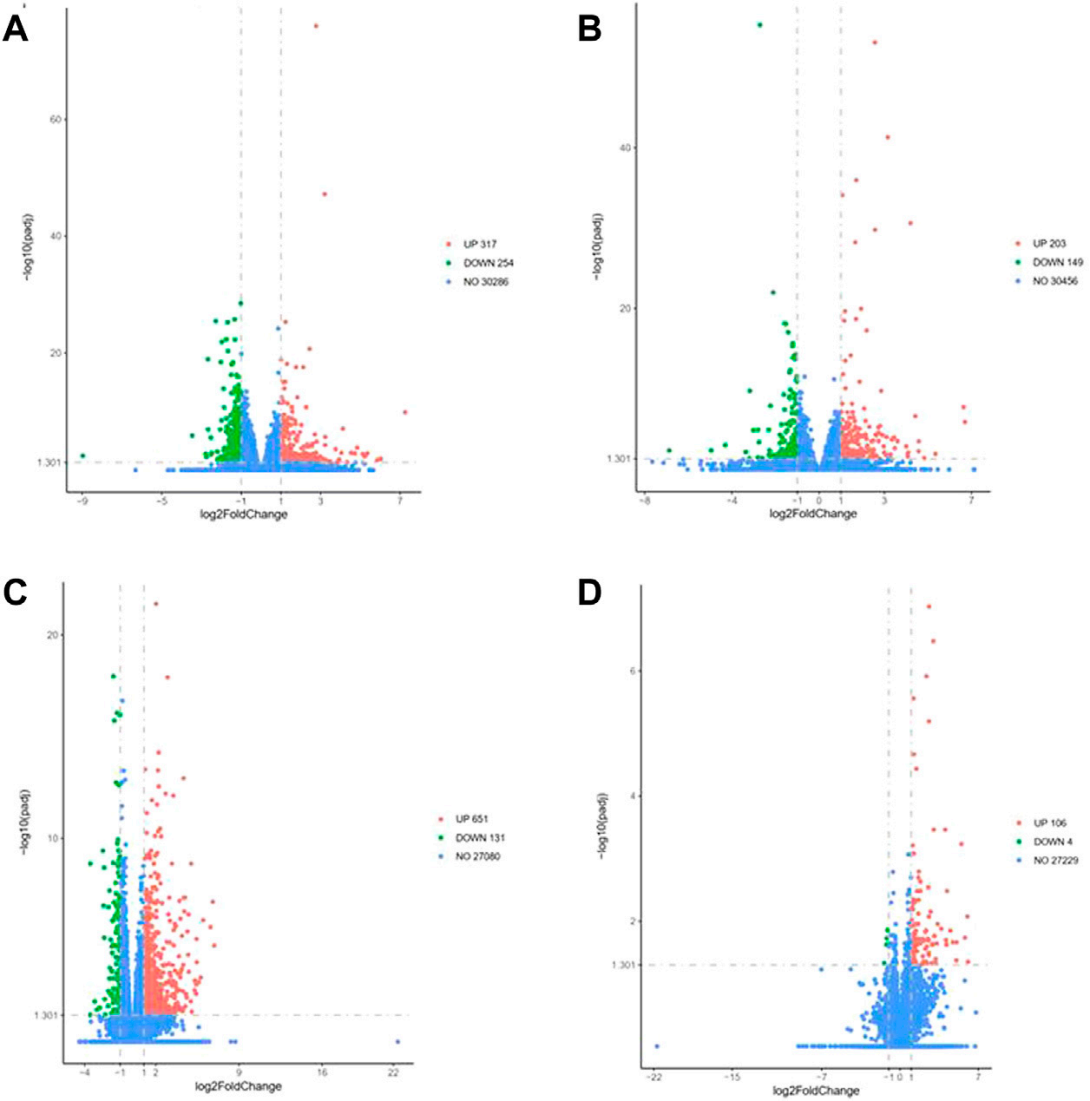
Volcano map of differentially expressed genes at 3 months in the **(A)** low-dose irradiation group, and **(B)** high-dose irradiation group, and at 6 months in the **(C)** low-dose irradiation group, and **(D)** high-dose irradiation group (|log2(Fold Change)| > 1 & Padj <0.05). Thirty mice were randomized into 3 groups: control group, low-dose irradiation (16 Gy) group and high-dose irradiation (20 Gy) group. Lung tissues were harvested at 3 and 6 months after radiation to perform RNA sequencing.

To understand the differences in the expression of RIPF, we conducted RNA sequencing and compared the mRNA expression in the irradiated mice with those in the control group. Three months after radiation therapy, in the low-dose irradiation group, 317 mRNAs were upregulated and 254 were downregulated significantly (Padj <0.05, fold change ≥2), compared with the control group. In the high-dose irradiation group, 203 mRNAs were upregulated and 149 were downregulated significantly. The most upregulated mRNA in the low-dose irradiation group, after 3 months, was Gm10800, with a 153-fold change, while the most downregulated mRNA was Igkv4-74, with a 0.002-fold change. The most upregulated mRNA in the high-dose irradiation group was Ighv1-54, with a 103-fold change, and the most downregulated one was Nppa, with a 0.008-fold change. Six months after radiation therapy, in the low-dose irradiation group, 651 mRNAs were upregulated and 131 were downregulated significantly (Padj <0.05, fold change ≥2). In the high-dose irradiation group, 106 mRNAs were upregulated and 4 downregulated significantly. The most upregulated mRNA in the low-dose irradiation group after 6 months was Awat1, with a 121-fold change, and the most downregulated mRNA was Gm8941, with a 0.009-fold change. Igkv4-69 was the most upregulated mRNA in the high-dose irradiation group, with a 68-fold change, while Gm16499 was the most downregulated, with a 0.4-fold change. Volcano plots of the mRNAs in the 3 groups at the 2 time points depict their apparent variations (Figure 3). Corresponding mRNAs related to different doses or time points are shown in supplementary materials. The top five upregulated and downregulated mRNAs, which may serve as potential biomarkers for RIPF, are also illustrated in Tables 1, 2.

**TABLE 1:** Top 5 Significantly Up and Downregulated mRNAs in the radiation groups at 3 months.

Gene name	log2FoldChange	Regulation	Group (Gy)
*GM10800*	7.259828	up	16
*IGHV1-58*	6.024468	up	16
*CAPN11*	5.849797	up	16
*SERPINB11*	5.350875	up	16
*CWH43*	5.214893	up	16
*IGKV4-74*	−8.97044	down	16
*GM47732*	−3.46232	down	16
*MARCO*	−2.77704	down	16
*GM46209*	−2.74462	down	16
*ANKRD63*	−2.67242	down	16
*IGHV1-54*	6.693227	up	20
*GM10800*	6.630226	up	20
*IGKV4-63*	5.338763	up	20
*IGHV1-75*	4.822869	up	20
*IGKV1-135*	4.575986	up	20
*NPPA*	−6.8779	down	20
*FITM1*	−4.94974	down	20
*ATP6V1B1*	−4.30951	down	20
*SLC7A14*	−3.36697	down	20
*KCNA1*	−3.20163	down	20

**TABLE 2:** Top 5 Significantly Up- and Downregulated mRNAs in the radiation groups at 6 months.

Gene name	log2FoldChange	Regulation	Group (Gy)
*AWAT1*	6.914182	up	16
*MRGPRB2*	6.797883	up	16
*SERPINA1D*	6.602139	up	16
*MRGPRX2*	5.98075	up	16
*GM12002*	5.777894	up	16
*GM8941*	−3.52852	down	16
*ANKRD63*	−3.52434	down	16
*GM22574*	−3.14978	down	16
*GM45444*	−2.9568	down	16
*GM26674*	−2.7308	down	16
*IGKV4-69*	6.080729	up	20
*MRGPRB2*	6.028619	up	20
*SERPINA1D*	5.813064	up	20
*IGHV3-1*	5.492749	up	20
*IGKV12-38*	5.062207	up	20
*GM16499*	−1.39981	down	20
*JPH1*	−1.2144	down	20
*GLP1R*	−1.1775	down	20
*GM49326*	−1.1194	down	20

### 3.5 Enrichment analysis of DEGs

GO, KEGG, and Reactome enrichment analyses were performed to further investigate the potential functions of these DEGs, and the pathways involved with them.

**FIGURE 4:**
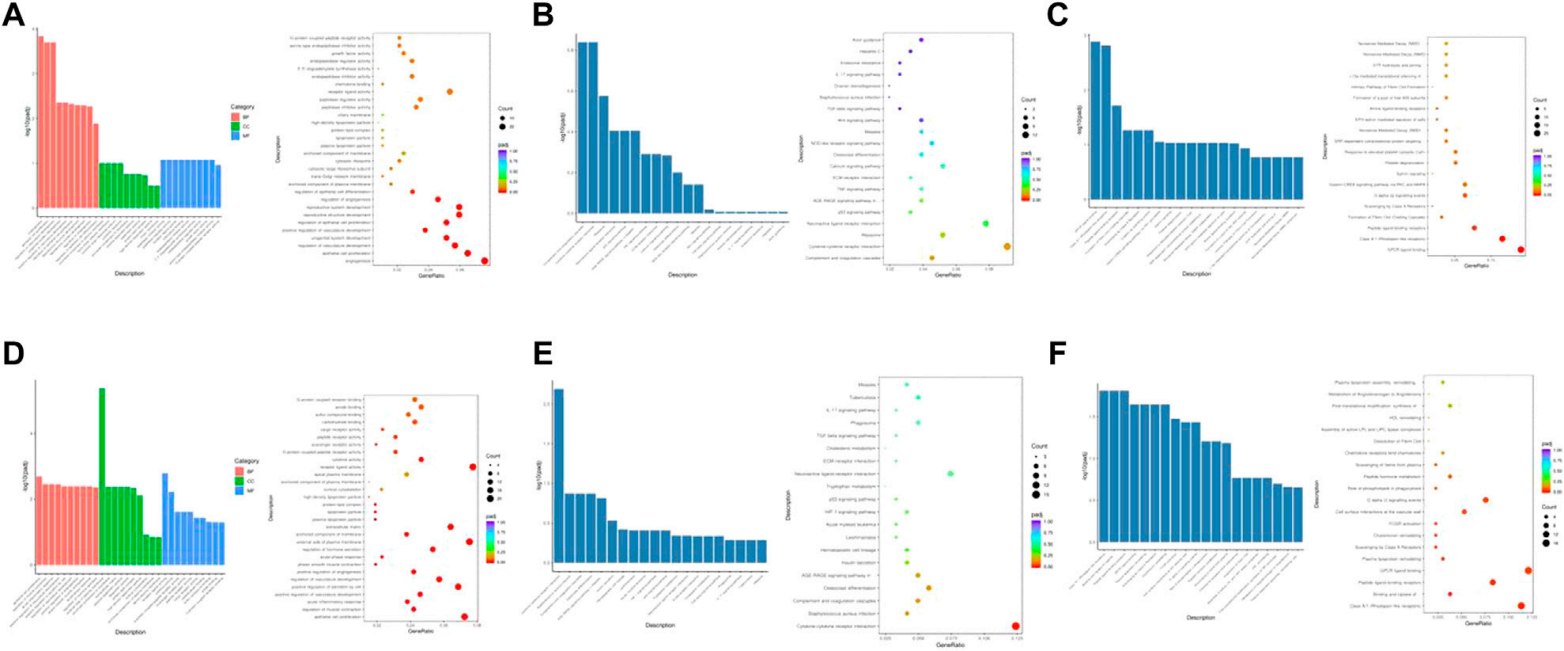
Enrichment analyses of differentially expressed genes at 3 months after radiation. **(A)** GO function enrichment analysis, **(B)** KEGG pathway analysis, and **(C)** Reactome enrichment analysis of differentially expressed mRNAs in the low-dose irradiation group after 3 months **(D)** GO function enrichment analysis, **(E)** KEGG pathway analysis, and **(F)** Reactome enrichment analysis of differentially expressed mRNAs in the high-dose irradiation group after 3 months.

Three months after radiation therapy, as shown in Figure 4A, angiogenesis, epithelial cell proliferation, and regulation of vasculature development were the main functions associated with the DGEs in the low-dose irradiation group. Epithelial cell proliferation, extracellular matrix, external side of the plasma membrane, and receptor ligand activity were the main functions in the high-dose irradiation group (Figure 4D). For KEGG pathway analysis, the DEGs were mainly enriched in the complement and coagulation cascades, cytokine-cytokine receptor interactions, ribosome, TNF signaling pathway, and NOD-like receptor signaling pathway in the low-dose irradiation group (Figure 4B). Cytokine-cytokine receptor interaction, *Staphylococcus aureus* infection, complement and coagulation cascades, and the HIF-1 signaling pathway were enriched in the high-dose irradiation group (Figure 4E). For Reactome enrichment analysis, the DEGs were mainly enriched in the GPCR ligand binding and class A/1 (rhodopsin-like receptors) signaling pathways in the low-dose irradiation group (Figure 4C). Class A/1 (rhodopsin-like receptors) and the binding and uptake of ligands by scavenger receptors were mainly enriched in the high-dose irradiation group (Figure 4F).

**FIGURE 5:**
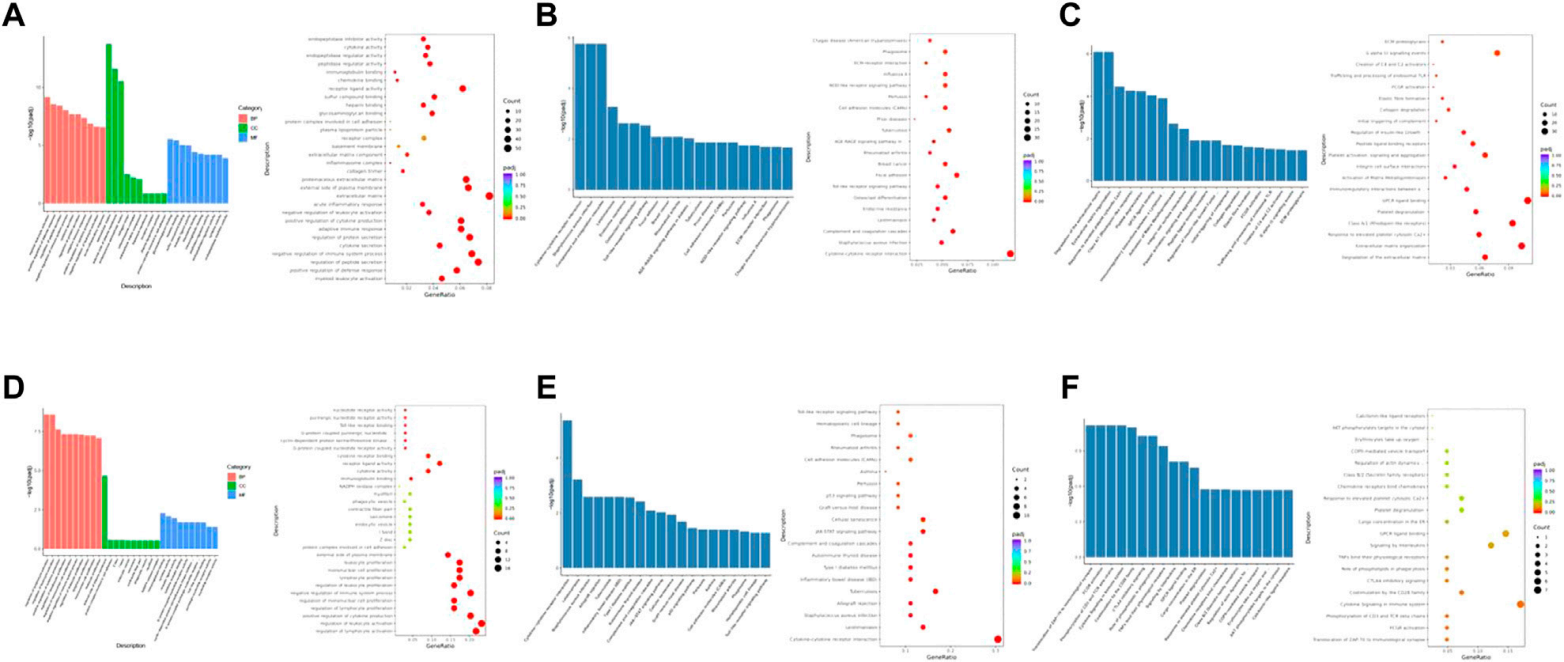
Enrichment analyses of differentially expressed genes at 6 months after radiation. **(A)** GO function enrichment analysis, **(B)** KEGG pathway analysis, and **(C)** Reactome enrichment analysis of differentially expressed mRNAs in the low-dose irradiation group after 6 months **(D)** GO function enrichment analysis, **(E)** KEGG pathway analysis, and **(F)** Reactome enrichment analysis of differentially expressed mRNAs in the high-dose irradiation group after 6 months. BP, Biological Process; CC, Cellular Component; MF, Molecular Function.

Six months after radiation therapy, as shown in Figure 5A, myeloid leukocyte activation, extracellular matrix, and glycosaminoglycan binding were the main functions associated with dysregulated genes in the low-dose irradiation group. Regulation of lymphocyte activation, regulation of leukocyte activation, regulation of lymphocyte proliferation, lymphocyte proliferation, mononuclear cell proliferation, immunoglobulin binding, and several immune system-associated functions were significantly enriched in the high-dose irradiation group (Figure 5D). KEGG pathway analysis showed that the DEGs were mainly enriched in cytokine-cytokine receptor interaction, *S. aureus* infection, complement and coagulation cascades, toll-like receptor signaling pathway, and NOD-like receptor signaling pathway in the low-dose irradiation group (Figure 5B). Leishmaniasis, *S. aureus* infection, tuberculosis, and cellular senescence were mainly enriched in the high-dose irradiation group (Figure 5E). For Reactome enrichment analysis, the differentially expressed genes were mainly enriched in degradation of the extracellular matrix, extracellular matrix organization, and response to elevated platelet cytosolic Ca2+ in the low-dose irradiation group (Figure 5C). Translocation of ZAP-70 to the immunological synapse, FCGR activation, phosphorylation of CD3 and TCR zeta chains, and cytokine signaling in the immune system were enriched in the high-dose irradiation group (Figure 5F).

### 3.6 Many radiation responsive genes in RIPF are immune system related

**FIGURE 6:**
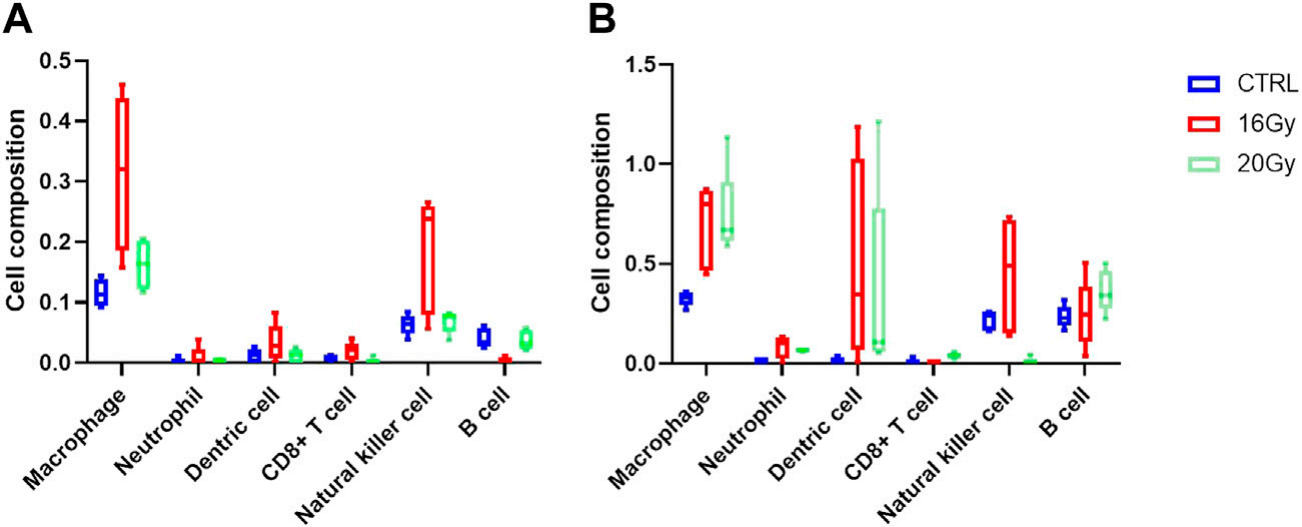
Immune cell infiltration estimation. Thirty mice were randomized into 3 groups: control group, low-dose irradiation (16 Gy) group and high-dose irradiation (20 Gy) group. Lung tissues were harvested at 3 and 6 months after radiation. **(A)** Relative proportion of estimated immune cell subsets in each group at 3 months **(B)** Relative proportion of estimated immune cell subsets in each group at 6 months. CTRL, control group.

The enrichment analyses of DEGs along with histological examination showed that immune cells may play a significant role in the development of RIPF. To explore the composition of the immune cells under different RIPF conditions, CIBERSORT, a tool for estimating the fractions of the different immune cell types, was used. The general immune cell abundance in each group is shown in Figure 6. We confirmed a dramatic increase in the number of macrophages in the irradiated groups at 3 months (Figure 6A). At 6 months, there was still an evident increase in macrophages, while the infiltration of B cells grew, and was more than control in 20 Gy group (Figure 6B).

## 4 Discussion

Our study identified radiation-responsive genes and analyzed the DEGs based on functional and pathway enrichment. We found several main responsive genes associated pathways that are significant for the prevention of RIPF as well as the improvement of radiation therapy.

Radiotherapy is indispensable for the treatment of thoracic malignancies including lung cancer, esophageal cancer, breast cancer, thymic cancer, and lymphoma. Despite advances in radiation therapy, 2%–37% of the patients with lung and breast cancer who receive thoracic radiotherapy can develop radiation-induced pneumonitis and have a higher risk of developing RIPF for months to years after radiation therapy (Tyldesley et al., 2001; Carver et al., 2007). Cella et al. reported that among 115 patients with Hodgkin lymphoma, who received 3D conformal radiotherapy, the incidence of RIPF was 16%, of which 8% cases were accompanied by obvious symptoms (Cella et al., 2014). The pathological changes in radiation-induced lung injury can be divided into exudation, granulation tissue growth, fibrous proliferation, and collagen formation. This four-stage pathological process reflects the development of RIPF due to radiation-induced pneumonia (Bai et al., 1995). RIPF includes a heterogeneous group of lung disorders characterized by progressive and irreversible destruction of lung architecture and disruption of gaseous exchange. The pathological mechanism of RIPF can be divided into three categories: 1) cellular and molecular regulation, including the interaction of type II alveolar epithelial cells, vascular endothelial cells, fibroblasts, alveolar macrophages, basement membrane cells, and blood cells; 2) autoimmune response; and 3) genetics and gene theory (Kong et al., 2005; He et al., 2019; Jin et al., 2019). Typical clinical symptoms of pulmonary fibrosis include increasing dyspnea, deterioration of lung function, aggravation of interstitial edema, and eventually, respiratory failure. Although anti-inflammatory therapy based on steroidal hormones has been widely used to control acute radiation pneumonia, there is no effective treatment approved for RIPF (Williams et al., 2010; Arroyo-Hernández et al., 2021). Therefore, the development of effective treatments is crucial to delay, inhibit, and reverse RIPF.

The differentially expressed mRNAs were implicated in several biological functions, including angiogenesis, epithelial cell proliferation, and extracellular matrix in the irradiated groups at 3 months. The associated significantly dysregulated genes included *IGHV1-58, CWH43, IGHV1-54, IGKV4-63, IGHV1-75,* and *IGKV1-135*. Imbalanced angiogenesis and abnormal blood vessel function is a common pathological process in respiratory disorders, including idiopathic pulmonary fibrosis, pulmonary arterial hypertension, chronic obstructive pulmonary disease, hepatopulmonary syndrome, and cigarette associated lung injury (Hanumegowda et al., 2012; Raevens et al., 2018; Eldridge and Wagner, 2019; Yang et al., 2021; Elamaa et al., 2022). Angiogenesis was also observed during the early to middle stages of RIPF. Lungs are characterized by double vasculature; therefore, the vascular system is vital to pulmonary physiology. Vascular endothelial growth factor (VEGF) is an important angiogenic factor. In addition to stimulating angiogenesis, VEGF-A can also influence lung development and homeostasis. VEGF-A stimulates alveolar type II cell growth (Brown et al., 2001; Varet et al., 2010) and surfactant production (Compernolle et al., 2002) in lung repair, following injury. It has also been reported that overexpression of TGF-β1 induces rat peritoneal fibrosis, accompanied by angiogenesis, through the induction of VEGF-A production in mesothelial cells (Margetts et al., 2001). Additionally, in bleomycin-induced pulmonary fibrosis, commonly used to elucidate the mechanism of pulmonary fibrosis, an increase in VEGF-A and CD31 expression has been found in fibrotic regions (Amano et al., 2019). Studies have also suggested that treatment targeting VEGF significantly attenuates bleomycin-induced pulmonary fibrosis *in vivo* (Hamada et al., 2005; Wan et al., 2013; Iyer et al., 2015; Laddha and Kulkarni, 2019). Whether anti-angiogenesis therapy can attenuate or cure RIPF remains to be verified.

Pathway enrichment analysis revealed that DEGs at 3 months were involved in the NOD-like receptor, TNF, and HIF-1 signaling pathways, all of which are injury- and inflammation-related pathways. NOD-like receptor protein 1 is called the first inflammasome and exerts its biological activity as an inflammasome complex (Chavarría-Smith and Vance, 2015). Inflammasomes are widely expressed by immune and non-immune cells, including monocytes/macrophages, B cells, T cells, DCs, fibroblasts/myofibroblasts, endothelial cells, and parenchymal cells (Moulton and Tsokos, 2015; Durai and Murphy, 2016; Rawlings et al., 2017; Lang et al., 2018; Le Bras, 2018). NOD-like receptor protein 1 mediates myocardial fibrogenesis in mice *via* the mitogen-activated protein kinase (MAPK), nuclear factor-κB (NF-κB), and TGF-β/Smad (Zong et al., 2018a; Zong et al., 2018b). In addition, previous studies have found that NLRP3 inflammasome promotes epithelial-mesenchymal transition, leading to pulmonary fibrosis (Sayan and Mossman, 2016; Lv et al., 2018). It has also been demonstrated that the pro-inflammatory cytokine TNF-α induces NF-κB activation, promoting myofibroblast differentiation of the lung resident mesenchymal stem cells, and exacerbates bleomycin-induced pulmonary fibrosis (Hou et al., 2018). Based on this and our data, it can be assumed that these pathways may also play important roles in the development of RIPF.

We observed that at 6 months, immune-associated functions were significantly enriched, including myeloid leukocyte activation in the low-dose irradiation group and lymphocyte activation, lymphocyte proliferation, mononuclear cell proliferation, and immunoglobulin binding in the high-dose irradiation group. Some of the significantly dysregulated genes included *AWAT1, MRGPRB2, IGKV4-69, IGHV3-1,* and *IGKV12-38*. To gain insight into the distribution of immune cells, infiltration analysis was performed. It was observed that macrophages dominated at all times. The number of B cells increased with time and seemed to be related to the irradiation dose. Macrophages regulate tissue regeneration after injury. They may worsen tissue injury by producing reactive oxygen species and toxic mediators that disrupt cell metabolism, induce apoptosis, and exacerbate ischemic injury (Oishi and Manabe, 2018). On the other hand, they produce a variety of growth factors such as VEGF-α, IGF-1, TGF-β, and Wnt proteins, which can regulate proliferation of epithelial and endothelial cells, activation of myofibroblasts, and differentiation of stem and tissue progenitor cells (Rappolee et al., 1988; Willenborg et al., 2012). However, dysregulated macrophage function may impair wound healing and lead to fibrosis (Mosser and Edwards, 2008; Vannella et al., 2014; Vannella and Wynn, 2017). B cell activation has been increasingly reported to be linked to fibrotic lung diseases. Activation of B cells by pattern recognition receptors induces the release of inflammatory cytokines, chemokines, and metalloproteases, which play an important role in the pathophysiology of idiopathic pulmonary fibrosis (Ali et al., 2017a; Ali et al., 2017b). Evidence of B cell activation in patients with RIPF is relatively low. In this study, with RIPF progression, the role of B cells seemed to manifest progressively as well.

CD3 and TCR zeta chains, cytokine signaling in the immune system, and cellular senescence in the radiation groups were also enriched in the pathway analyses at 6 months. The former two are consistent with the functional analyses. An increasing body of evidence suggests that induction of senescence by radiation may play an important role in RIPF (Citrin et al., 2013; Schafer et al., 2017; He et al., 2019). It has been reported that the clearance of senescent cells using a senolytic agent, a small molecule that can selectively kill senescent cells, has the potential to be developed as a novel therapeutic strategy for RIPF (Justice et al., 2019). Our data are in line with those of previous studies, and notably, cellular senescence was also observed in the late stages of RIPF.

Using RNA sequencing, we identified a large number of mRNAs at different stages of RIPF. These mRNAs were involved in cellular metabolism, cell injury, extracellular matrix function, epithelial cell proliferation, and immune system activation and regulation. Our results provide better understanding of the mechanism involved in the development of RIPF, which may be potential targets for preventing and treating RIPF.

## Data Availability

The data presented in the study are deposited in the SRA repository, accession number PRJNA887533.
