# Supplementary material for: The mapping of mRNA alterations elucidates the etiology of radiation-induced pulmonary fibrosis
Source: Front Genet. 2022 Oct 24;13:999127. doi: 10.3389/fgene.2022.999127 (PMC9638132; doi:10.3389/fgene.2022.999127)
Supplement: Supplementary file 1 [file Presentation1.PPTX]

## Slide 1
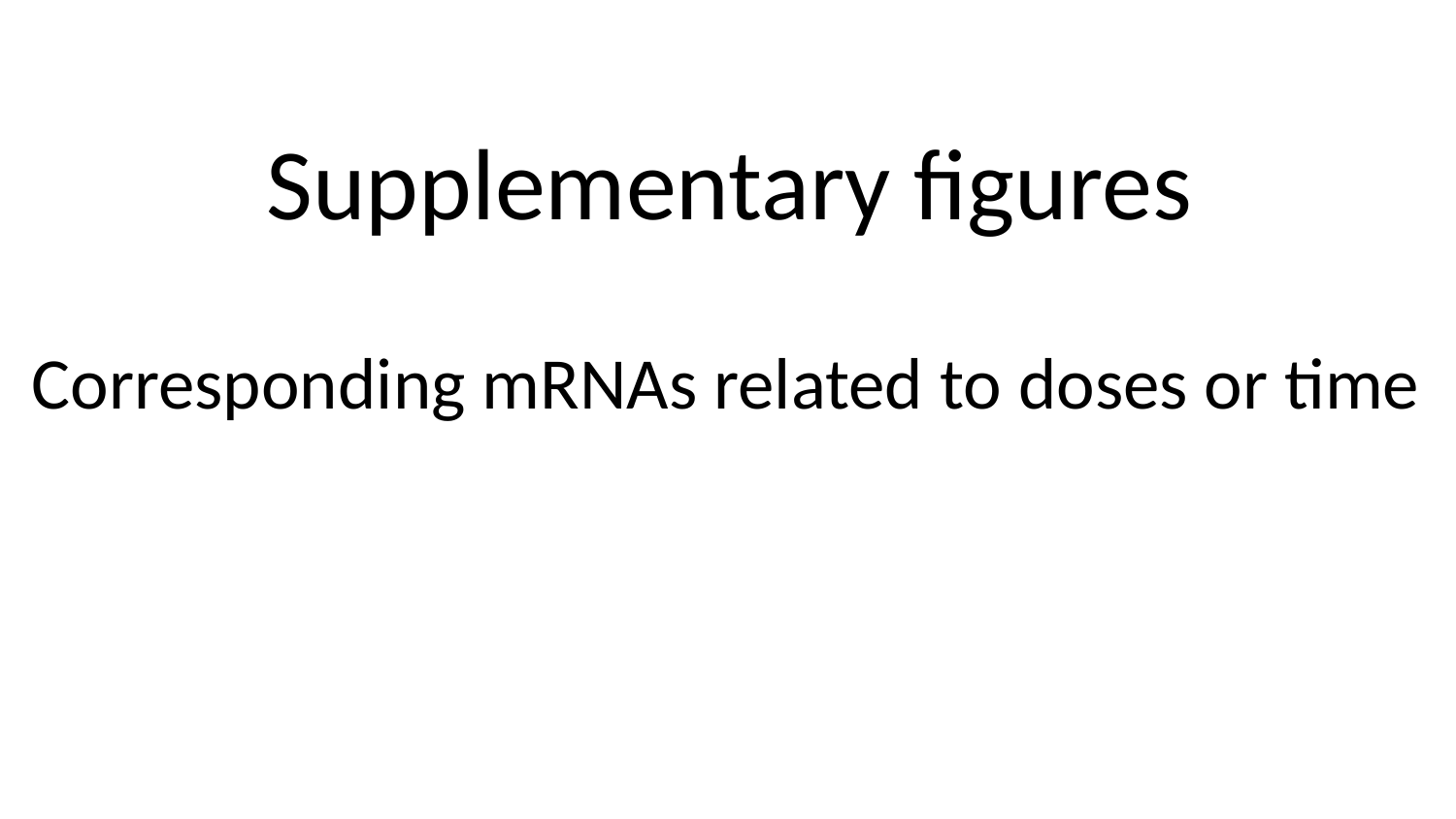

# Supplementary figures
Corresponding mRNAs related to doses or time

## Slide 2
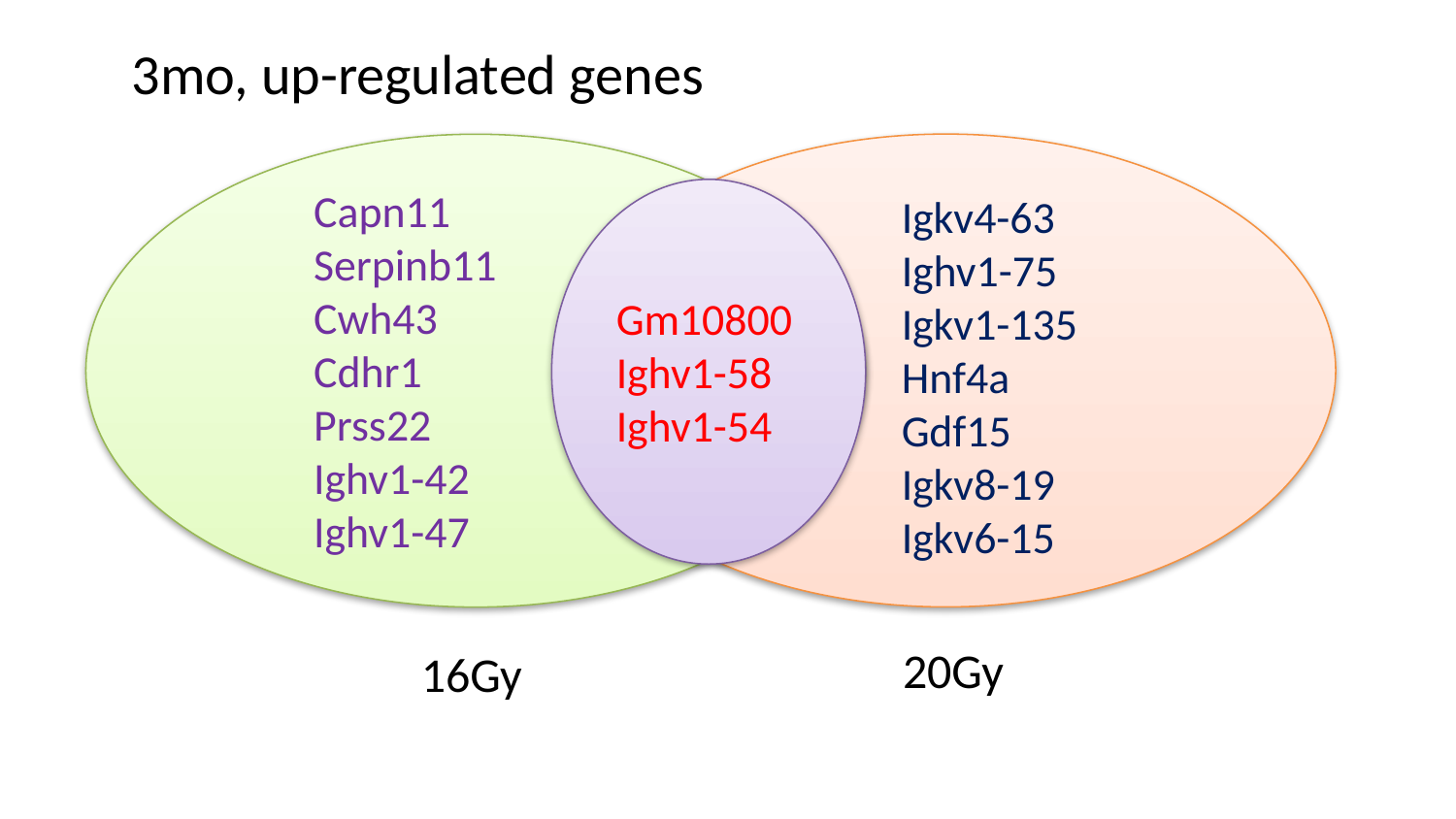

3mo, up-regulated genes
Capn11
Serpinb11
Cwh43
Cdhr1
Prss22
Ighv1-42
Ighv1-47
Igkv4-63
Ighv1-75
Igkv1-135
Hnf4a
Gdf15
Igkv8-19
Igkv6-15
Gm10800
Ighv1-58
Ighv1-54
20Gy
16Gy

## Slide 3
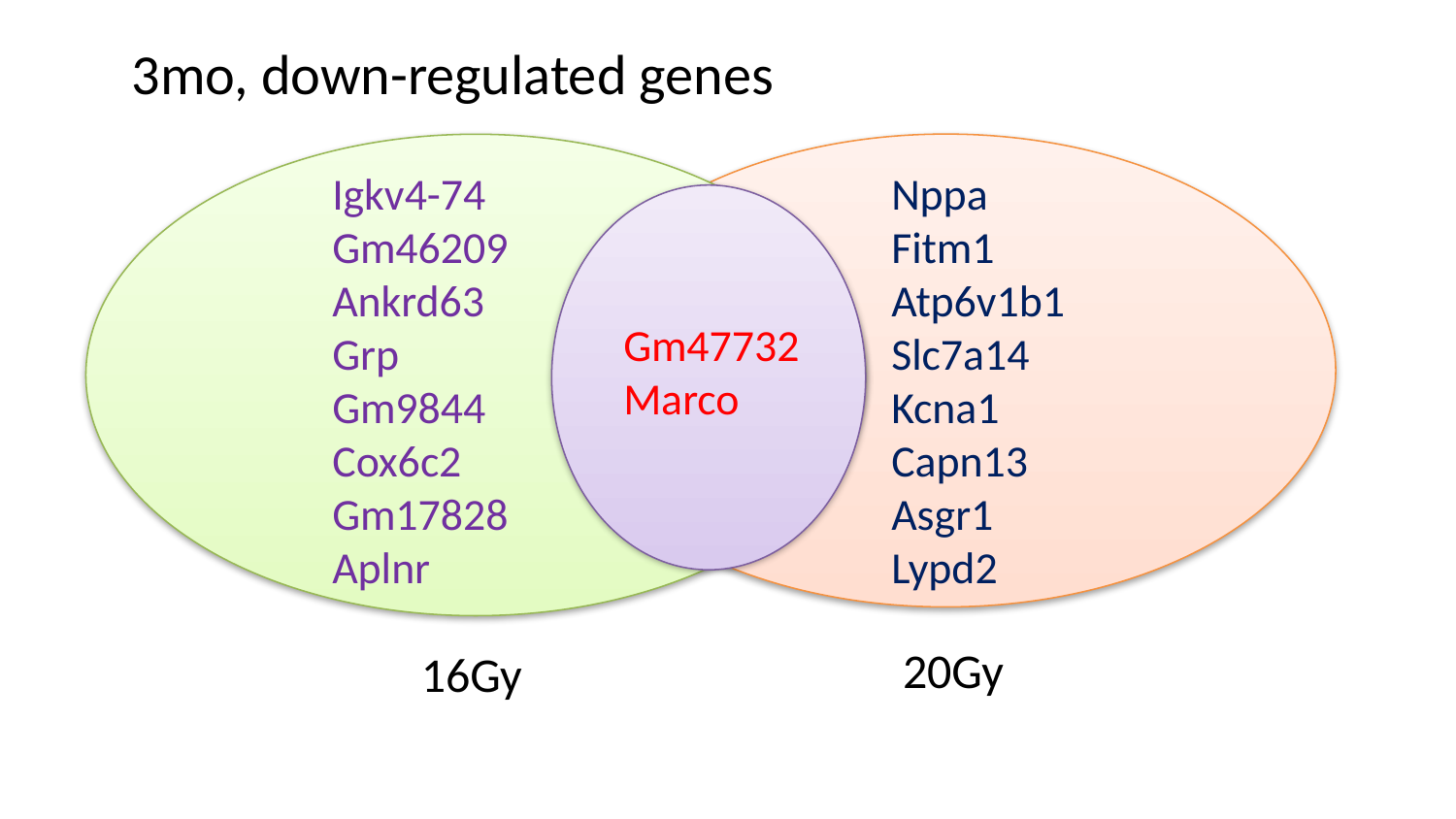

3mo, down-regulated genes
Igkv4-74
Gm46209
Ankrd63
Grp
Gm9844
Cox6c2
Gm17828
Aplnr
Nppa
Fitm1
Atp6v1b1
Slc7a14
Kcna1
Capn13
Asgr1
Lypd2
Gm47732
Marco
20Gy
16Gy

## Slide 4
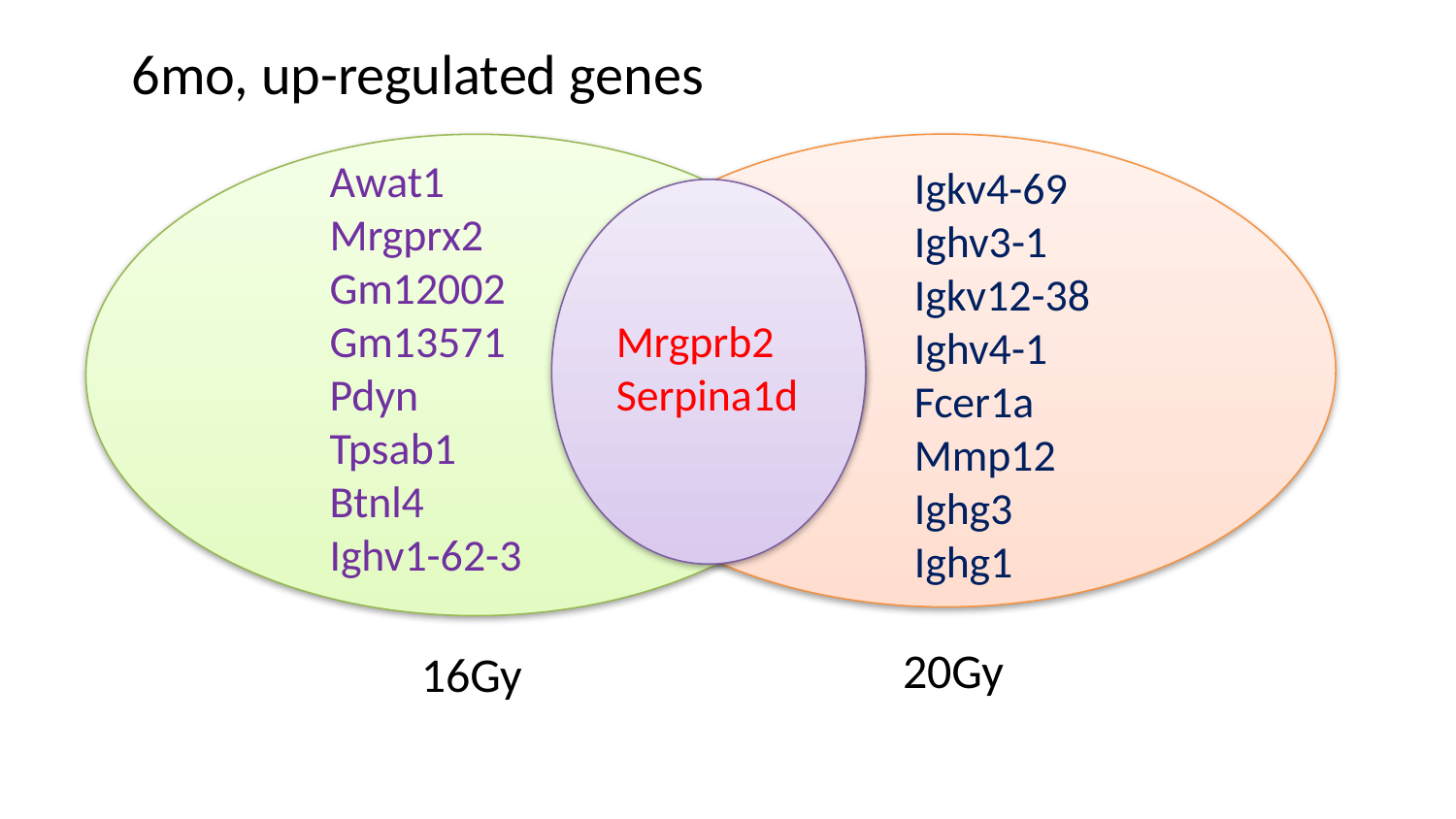

6mo, up-regulated genes
Awat1
Mrgprx2
Gm12002
Gm13571
Pdyn
Tpsab1
Btnl4
Ighv1-62-3
Igkv4-69
Ighv3-1
Igkv12-38
Ighv4-1
Fcer1a
Mmp12
Ighg3
Ighg1
Mrgprb2
Serpina1d
20Gy
16Gy

## Slide 5
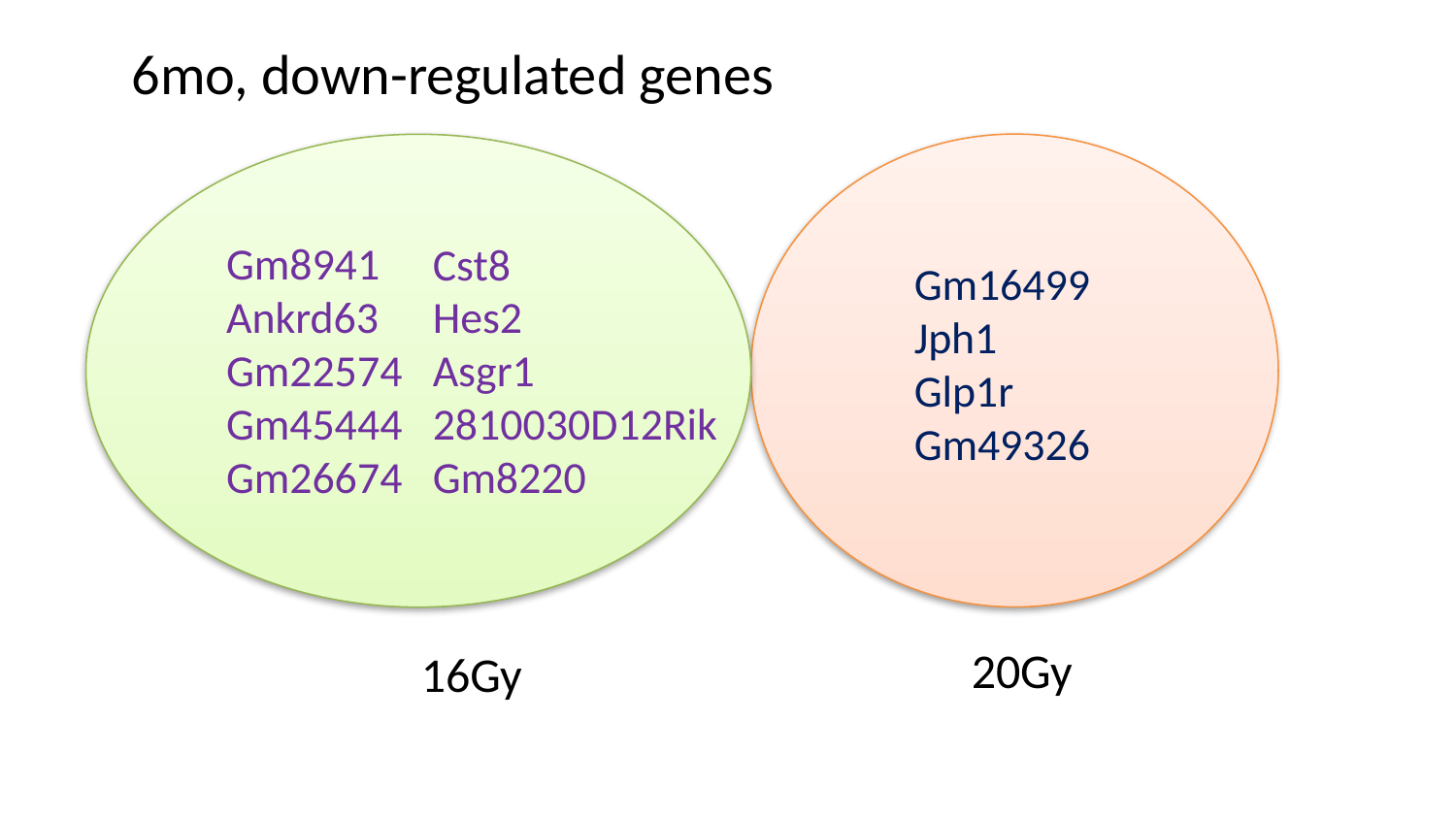

6mo, down-regulated genes
Gm8941
Ankrd63
Gm22574
Gm45444
Gm26674
Cst8
Hes2
Asgr1
2810030D12Rik
Gm8220
Gm16499
Jph1
Glp1r
Gm49326
20Gy
16Gy

## Slide 6
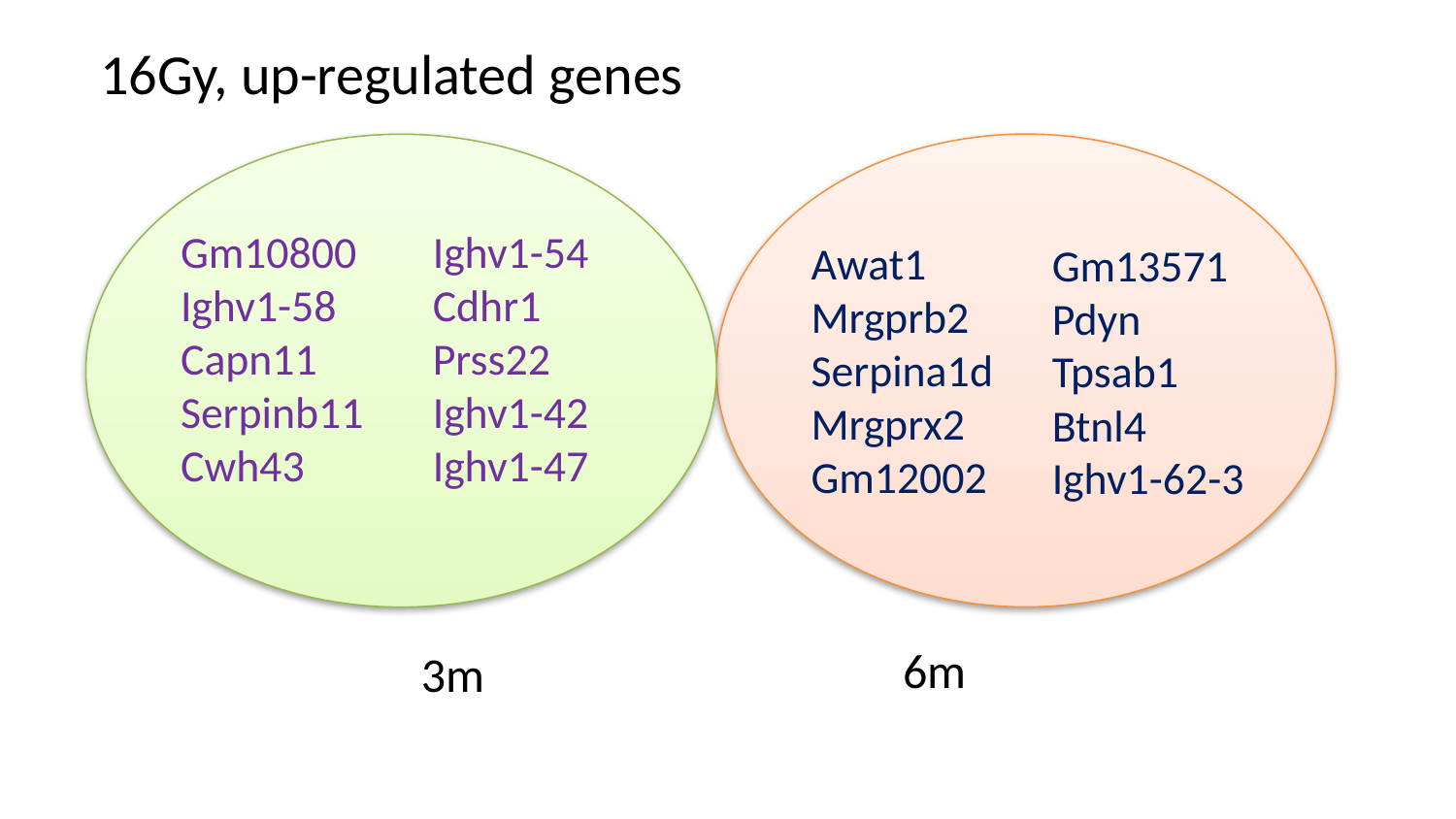

16Gy, up-regulated genes
Gm10800
Ighv1-58
Capn11
Serpinb11
Cwh43
Ighv1-54
Cdhr1
Prss22
Ighv1-42
Ighv1-47
Awat1
Mrgprb2
Serpina1d
Mrgprx2
Gm12002
Gm13571
Pdyn
Tpsab1
Btnl4
Ighv1-62-3
6m
3m

## Slide 7
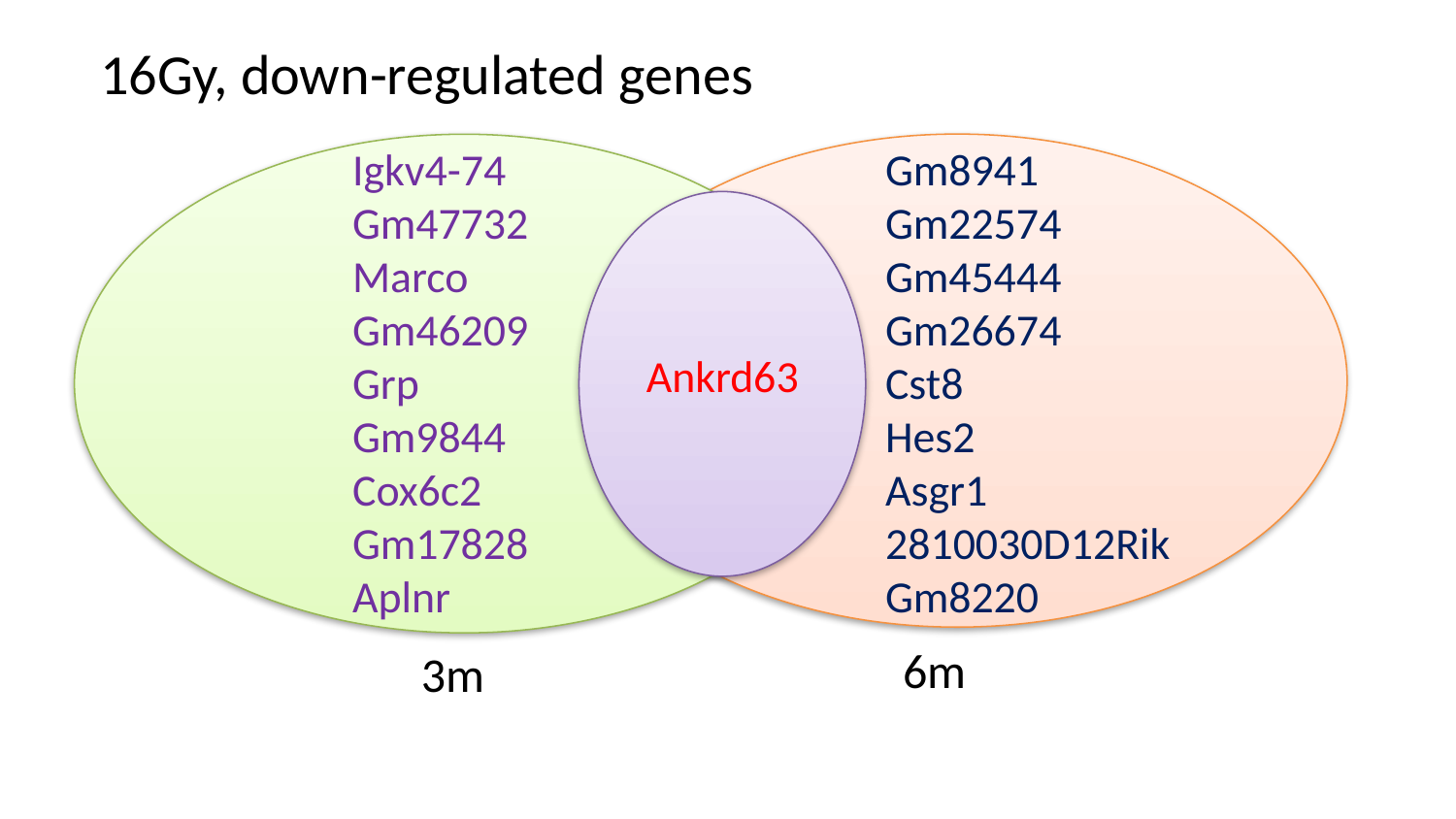

16Gy, down-regulated genes
Igkv4-74
Gm47732
Marco
Gm46209
Grp
Gm9844
Cox6c2
Gm17828
Aplnr
Gm8941
Gm22574
Gm45444
Gm26674
Cst8
Hes2
Asgr1
2810030D12Rik
Gm8220
Ankrd63
6m
3m

## Slide 8
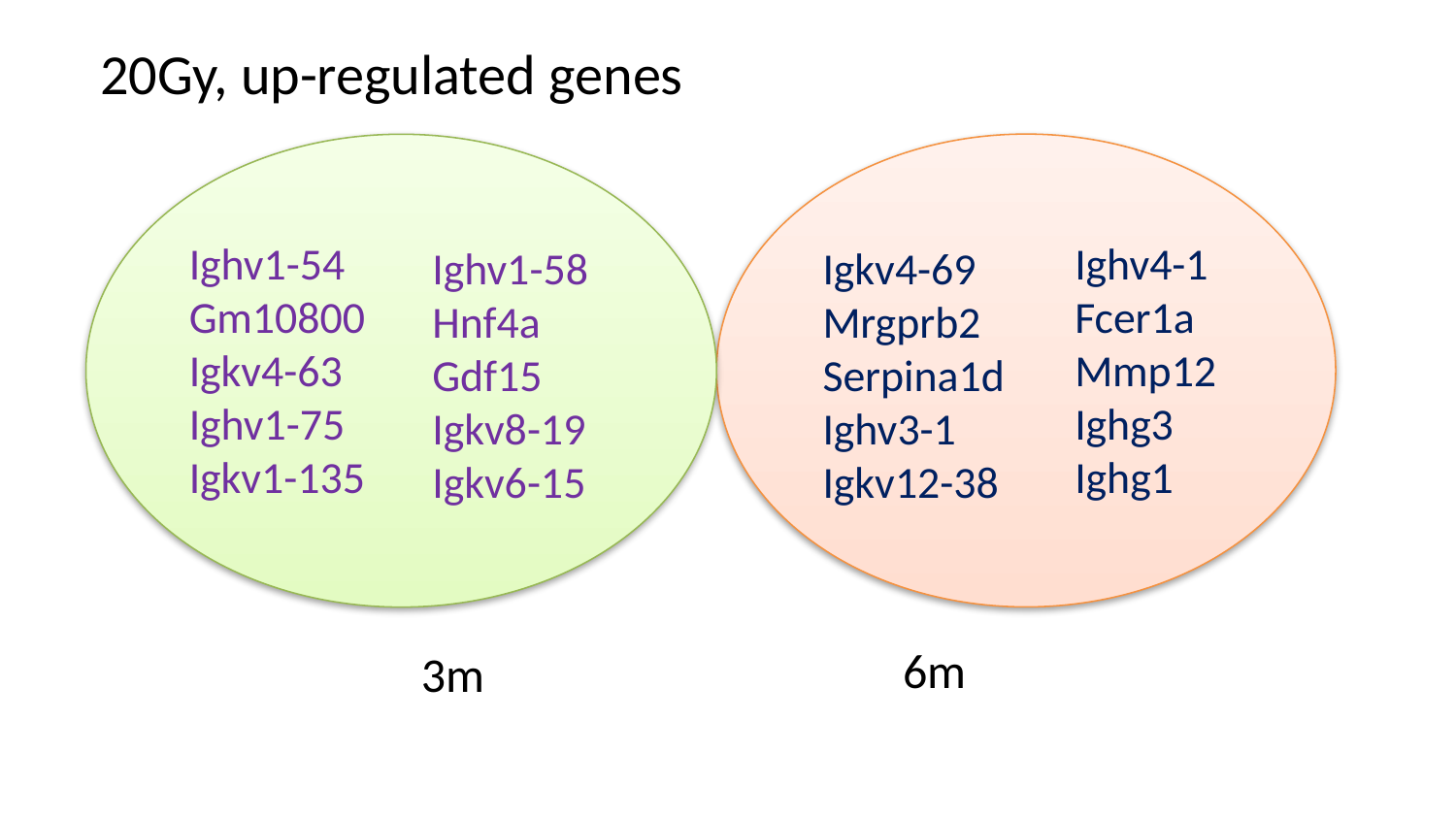

20Gy, up-regulated genes
Ighv1-54
Gm10800
Igkv4-63
Ighv1-75
Igkv1-135
Ighv4-1
Fcer1a
Mmp12
Ighg3
Ighg1
Ighv1-58
Hnf4a
Gdf15
Igkv8-19
Igkv6-15
Igkv4-69
Mrgprb2
Serpina1d
Ighv3-1
Igkv12-38
6m
3m

## Slide 9
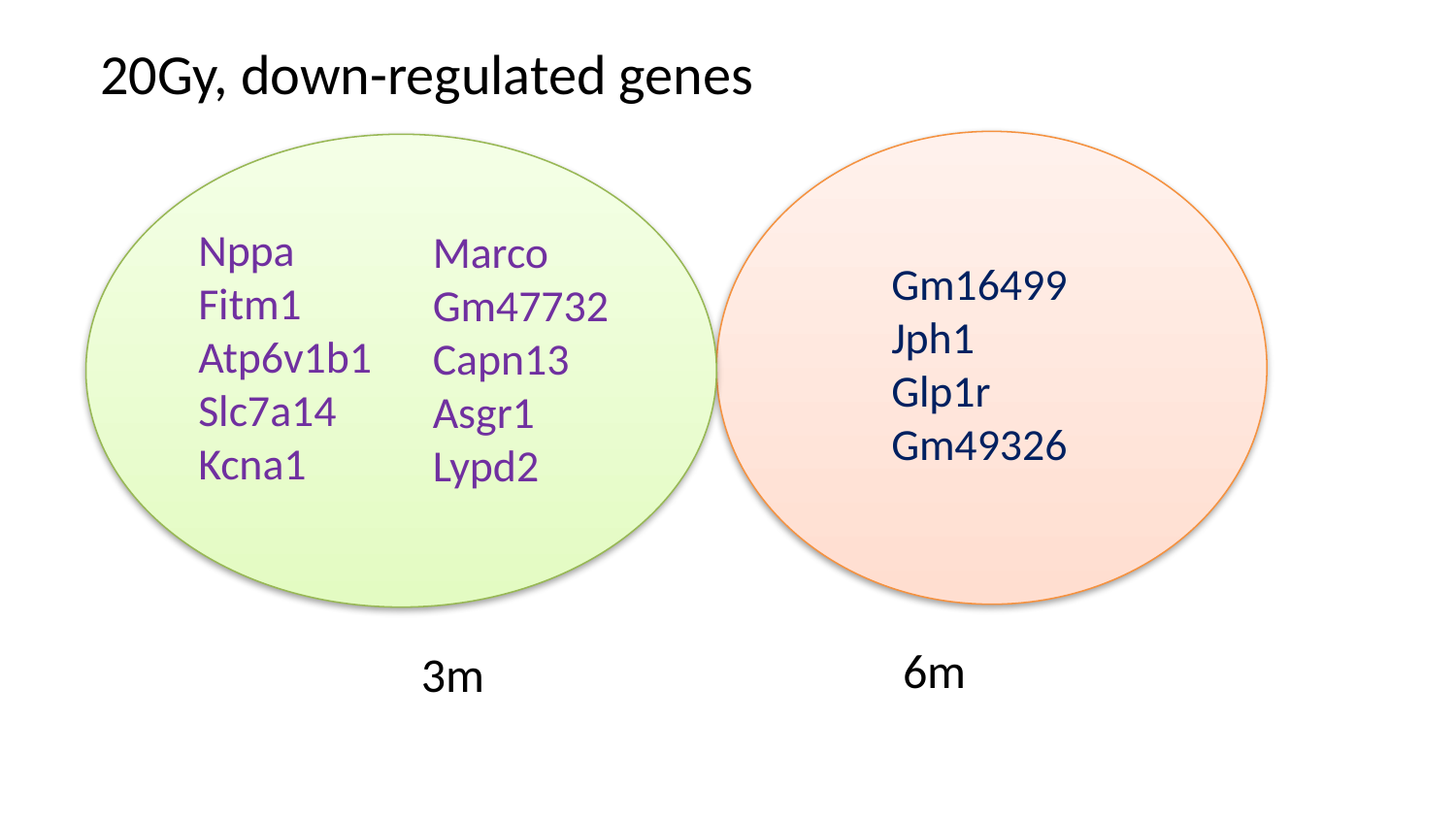

20Gy, down-regulated genes
Nppa
Fitm1
Atp6v1b1
Slc7a14
Kcna1
Marco
Gm47732
Capn13
Asgr1
Lypd2
Gm16499
Jph1
Glp1r
Gm49326
6m
3m
